# Stringent Base Specific and Optimization-Free Multiplex Mediator Probe ddPCR for the Quantification of Point Mutations in Circulating Tumor DNA

**DOI:** 10.3390/cancers13225742

**Published:** 2021-11-16

**Authors:** Franziska Schlenker, Elena Kipf, Max Deuter, Inga Höffkes, Michael Lehnert, Roland Zengerle, Felix von Stetten, Florian Scherer, Julius Wehrle, Nikolas von Bubnoff, Peter Juelg, Tobias Hutzenlaub, Nadine Borst

**Affiliations:** 1 Hahn-Schickard, Georges-Koehler-Allee 103, 79110 Freiburg, Germany; Franziska.schlenker@hahn-schickard.de (F.S.); elena.kipf@hahn-schickard.de (E.K.); inga.1999@gmx.de (I.H.); michael.lehnert@hahn-schickard.de (M.L.); Roland.Zengerle@Hahn-Schickard.de (R.Z.); felix.von.stetten@hahn-schickard.de (F.v.S.); Peter.Juelg@Hahn-Schickard.de (P.J.); Tobias.Hutzenlaub@Hahn-Schickard.de (T.H.); 2 Department of Medicine I, Medical Center—University of Freiburg, Faculty of Medicine, University of Freiburg, 79106 Freiburg, Germany; max.deuter@uniklinik-freiburg.de (M.D.); florian.scherer@uniklinik-freiburg.de (F.S.); julius.wehrle@uniklinik-freiburg.de (J.W.); Nikolas.vonBubnoff@uksh.de (N.v.B.); 3 Laboratory for MEMS Applications, IMTEK—Department of Microsystems Engineering, University of Freiburg, Georges-Koehler-Allee 103, 79110 Freiburg, Germany; 4 Department of Hematology and Oncology, Campus Lübeck, University Hospital Schleswig-Holstein, 23562 Lübeck, Germany

**Keywords:** mediator probe PCR, digital PCR, multiplex PCR, optimization-free, colorectal carcinoma, standardized universal reporter, treatment response, point mutations, circulating tumor DNA, ctDNA

## Abstract

**Simple Summary:**

Cancer treatment strategies and their follow-up monitoring are changing to personalized therapies, based on molecular genetic information from the individual person. Liquid biopsy, where this molecular information is derived from body fluids such as blood, has the potential to provide a systemic fingerprint of cancer dynamics, and, compared to tissue biopsy, is much less invasive for the patient. We used the previously published mediator probe PCR technology for liquid biopsy detection of several mutations in one reaction, so-called digital multiplex PCR. Quantification of point mutations in plasma eluates from follow-up patients using 4-plex digital assays showed a comparable performance to reference 2-plex assays. As a key feature, the presented multiplex assays require no laborious optimization as they use the same concentrations and cycling conditions for all targets. This allows for flexible design and interchangeable target panels, thus the assay is easily adaptable for individual patient monitoring and reduces sample consumption.

**Abstract:**

There is an increasing demand for optimization-free multiplex assays to rapidly establish comprehensive target panels for cancer monitoring by liquid biopsy. We present the mediator probe (MP) PCR for the quantification of the seven most frequent point mutations and corresponding wild types (*KRAS* and *BRAF*) in colorectal carcinoma. Standardized parameters for the digital assay were derived using design of experiments. Without further optimization, the limit of detection (LoD) was determined through spiking experiments with synthetic mutant DNA in human genomic DNA. The limit of blank (LoB) was measured in cfDNA plasma eluates from healthy volunteers. The 2-plex and 4-plex MP ddPCR assays showed a LoB of 0 copies/mL except for 4-plex *KRAS* G13D (9.82 copies/mL) and 4-plex *BRAF* V600E (16.29 copies/mL) and allele frequencies of 0.004% ≤ LoD ≤ 0.38% with R^2^ ≥ 0.98. The quantification of point mutations in patient plasma eluates (18 patients) during follow-up using the 4-plex MP ddPCR showed a comparable performance to the reference assays. The presented multiplex assays need no laborious optimization, as they use the same concentrations and cycling conditions for all targets. This facilitates assay certification, allows a fast and flexible design process, and is thus easily adaptable for individual patient monitoring.

## 1. Introduction

In recent years, the need for non-invasive early cancer detection for diagnosis, minimal residual disease monitoring, prediction, and prognosis has become more and more apparent. The molecular information serving as a systemic fingerprint of cancer dynamics is derived from body fluids through liquid biopsy [1,2,3,4,5,6]. Comprehensive target panels are necessary to monitor treatment response on a patient-individual level [7,8]. Ultrasensitive mutation detection in a high background of wild type DNA is typically achieved by using digital PCR (dPCR) [3,9,10,11]. The risk of false negative results due to the low amounts of DNA caused by splitting samples for multiple singleplex assays can be minimized by multiplex assays [12]. Therefore, multiplex assays are an indispensable part of patient monitoring in colorectal carcinoma (CRC) [13]. These assays must show high specificity and sensitivity, required for the quantification of low amounts of circulating tumor DNA (ctDNA) in the patient samples. However, in routine diagnostics, the complex optimization of current multiplex assays is not possible due to time and cost constraints. For example, locked nucleic acid (LNA) probes have a high specificity but require complex assay design, a cumbersome assay optimization process, and individual assay conditions (primer/probe/detergent concentrations, annealing time, annealing temperature, PCR cycles) [12]. In 2020, Hussung et al. [12,14] published several multiplex screening assays for *KRAS* and *NRAS* point mutations in pancreatic carcinoma using the LNA technique. However, the current multiplex LNA approach is more suitable for mutation screening as it still requires single-target assays for accurate mutation determination. 

In our work, we present multiplex assays for the quantification of point mutations using the mediator probe (MP) PCR technology. This comprises label-free MPs and standardized fluorogenic universal reporters (UR) so that the fluorescence signal generation is independent of the target DNA detection [15,16]. This mechanism features high specificity, especially for the detection of point mutations for the following reason: a unique feature of MP is that they are cleaved stringently base-specific and even a mismatch of one base pair will block signal generation at the UR [17]. A standard set of UR sequences for multiplex detection of different target panels was described by Wadle et al. [18]. Based on this standard set, guidelines were derived as to how to design UR with high signal-to-noise ratios (SNR) to improve the limit of detection and quantification in assays [15]. Moreover, Wadle et al. used a design of experiment (DOE) approach to successfully optimize real-time PCR probes [19]. The applicability of MP digital droplet (dd)PCR for the detection of *KRAS* point mutations was previously demonstrated in a publication on increasing the number of color channels by selective photobleaching [20]. Compared to a publication of Lee et al. [21] using the TOCE (Tagginge Oligonucleotide Cleavage and Extension) technology, the MP PCR technology features a temperature independent readout. This enables the application of the MP PCR technology for standard dPCR devices that have no possibility of heating during readout. 

In this work, we present MP ddPCR assays for the stringent base specific quantification of point mutations in ctDNA in an assay format, which does not require further optimization steps. All assays were based on a set of four standardized fluorogenic URs, fixed oligonucleotide concentrations, and cycling conditions independent of the point mutation. The standardized assay parameter was derived by one initial DOE and then allowed for a direct multiplexing of different target panels. Specificity to the point mutations is provided by label free target specific MPs. Aside from time saving aspects, this standardization of assay conditions is a big advantage when it comes to the clinical certification of assays. We show the assay validation of 2-plex and 4-plex MP ddPCR assays with patient samples in a clinical setting and compare them directly with a reference assay. With our guidelines, personalized and flexible multiplex panels no longer require compromises concerning sensitivity and specificity, and can thus be applied as a single-step molecular diagnostic tool for personalized cancer treatment. 

## 2. Materials and Methods

### 2.1. Chemicals and Oligonucleotides for MP ddPCR Assay

The oligonucleotides used for the MP ddPCR assay were purchased from biomers.net (Ulm, Germany) and stored at −20 °C. The sequence information for primers, MPs, and URs is included in Table S3. After assay development using DOE, the final concentration (f.c.) of the forward primer in the reaction mix was 500 nM, the f.c. of the reverse primer and the MP were both 1200 nM, and the f.c. of the UR was 240 nM. A 100 nM fluorescein sodium salt (high molecular grade, VWR, Bruchsal, Germany) was used for droplet detection. For DNA dilution and the no template control, a TE buffer at 1× concentration was prepared with 500 µL tris-EDTA buffer (100× concentrate, T9285-100 ML, Sigma-Aldrich, Taufkirchen, Germany) in 50 mL nuclease-free water (AM9937, Ambion by ThermoFisher scientific, Waltham, MA, USA). Synthetic DNA (gBlocks), serving as templates for the mutant assays, were purchased from Integrated DNA Technologies (IDT, Coralville, IA, USA). These were customized with a length of 131 base pairs each (except for *BRAF* V600E: 160 bp). The DNA sequences for each mutant sequence (*KRAS* G12D, G12V, G12A, G12S, G12C, G13D, and *BRAF* V600E) are included in the electronic Supplementary Materials (ESI; Tables S1 and S2). The DNA accession number for the *KRAS* sequences is N_000012.12 and for the *BRAF* sequence, it is N_000007.14. For the wild type detection, human genomic DNA (Roche, Basel, Switzerland) was digested with the restriction enzyme HaeIII (New England Biolabs, Ipswich, MA, USA) according to the manufacturer’s instruction.

### 2.2. Chemicals and Oligonucleotides for LNA PCR

The reference LNA assays [12,14] were performed on a Bio-Rad ddPCR system (QX 200). Therefore, the PCR reaction mix comprised the ddPCR Supermix for Probes (No dUTP) from Bio-Rad (Hercules, CA, USA), primers, probes, and synthetic DNA from IDT, and human genomic DNA from Roche. For DNA and oligonucleotide dilution as well as the no template control, nuclease free water from Ambion by ThermoFisher Scientific was used. The assay specific cycling parameters are detailed in Table S6.

### 2.3. MP ddPCR Assay Characterization

The MP ddPCR assays were performed on the Naica platform from Stilla Technologies (Villejuif, France). The chip preparation followed the manufacturer’s instructions. The cycling protocol for the 2-plex MP ddPCR assay included a 5 min hot start at 95 °C and 45 cycles of denaturation (15 s at 95 °C) and annealing/extension (60 s at 54 °C). For the 4-plex MP ddPCR assay, the same parameters were used, except for the annealing temperature. This was increased from 54 °C to 56 °C to increase the specificity. The readout of the 2-plex assays in the sapphire chips (Stilla Technologies) followed the manufacturer’s instructions for the Naica readout device, Prism3 (Stilla Technologies). As the Prism3 is limited to three fluorescence color channels, for the readout of the 4-plex MP ddPCR assay, a standard fluorescence microscope (LionheartLX, Biotek, Winooski, VT, USA) was used. The workflow for droplet readout with a customized chip holder and quantification with the LionheartLX microscope followed the previously published protocol [22].

The performances of the 2-plex and 4-plex MP ddPCR assays were characterized by serial dilution experiments. The DNA concentration for each mutant sequence (1011 DNA copies/reaction, 101 DNA copies/reaction, 10 DNA copies/reaction, and one DNA copies/reaction) was varied in a constant background of 12,000 copies/reaction wild type DNA. For the 2-plex ddPCR assays, the calculation of the quantified concentrations was performed using the CrystalMiner software (V. 2.4.0.3, Stilla Technologies, Villejuif, France), whereas the calculation for the 4-plex ddPCR assay was conducted manually using Poisson statistics in Excel (Microsoft). For both multiplex strategies, the linearity and determination coefficients (R^2^) were evaluated with OriginPro 2019.

The seven 2-plex MP ddPCR assays were each able to specifically quantify a mutant point mutation and its corresponding wild type sequence. To cover an increased spectrum of point mutations, the 2-plex assays were combined into three 4-plex MP ddPCR assays. These three combinations were named panels 1–3. The distribution of the mutant and wild type sequences can be found in Table S1 for the 2-plex MP ddPCR assay and Table S2 for the 4-plex MP ddPCR panels.

For the determination of the limit of blank (LoB), cfDNA eluates from healthy volunteers were extracted from plasma samples on a QIAsymphony (Qiagen, Hilden, Germany; extraction kit: DSP Circulating DNA Kit (Qiagen)). The eluates of four individuals were pooled and tested in 5 µL quadruplicates in the sapphire chip. The LoB is calculated according to Equation (1) in copies per mL plasma:

(1) LoB=mean+1.645×Stdev

Wherein the mean is the mean value of copies/mL plasma and Stdev is the standard deviation.

In addition to the LoB, the allele frequency (AF) is used to compare the assay characteristics of the MP ddPCR multiplex assays with the previously published LNA assays. Commonly, the lower limit of the clinically relevant AF is 0.1%. The AF is calculated from the copy number of the mutant target divided by the total number of copies in the reaction, following Equation (2):

(2) $$\frac{cps_{mutant\;target}}{cps{\;}_{mutant\;target}+cps_{wild\;type\;target}}\times 100\;\geq 0.1\%$$

For both the eluates from healthy volunteers and the plasma eluates from CRC patients, 2 mL plasma was used as the starting volume for the DNA extraction in the QIAsymphony SP. The subsequent elution volume was 60 µL. 

### 2.4. MP ddPCR Assay Validation with Clinical Samples

The 2-plex and 4-plex MP ddPCR assays were validated with patient samples in a clinical setting. For this, the same samples and the same eluate volumes (5 µL/reaction) were tested with the previously published reference assays (2-plex LNA assays in Bio-Rad system) and with the 2-plex and 4-plex MP ddPCR assays on the Stilla Naica platform. The assay validation included 18 clinical samples from seven patients with different point mutations and sampling time points. The AF to be measured in the clinical samples was intended for the following mutations: *KRAS* G12D, G12C, G13D, and *BRAF* V600E. 

### 2.5. Principle for Point Mutation Detection by Mediator Probe PCR

**Figure 1:**
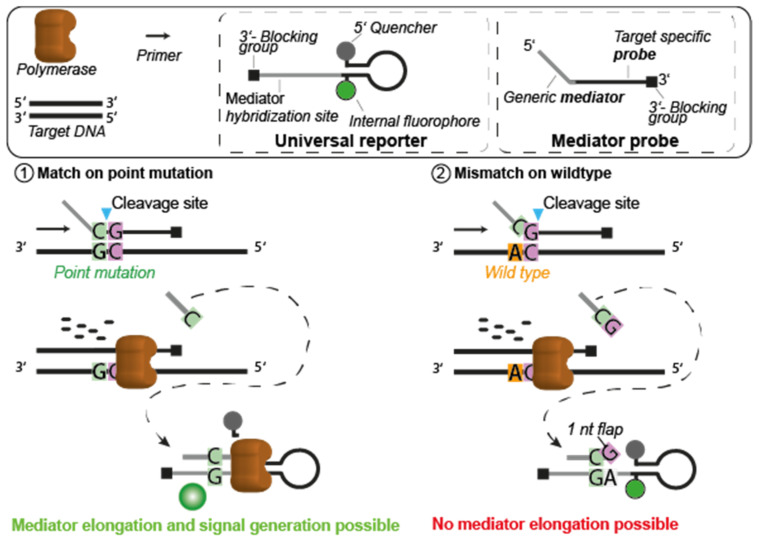
Principle for point mutation detection by MP PCR. The MP comprises a target specific probe and a generic mediator. During primer elongation, the mediator is cleaved by the polymerase and diffuses to the signal generating molecule, the UR oligonucleotide. There, the mediator can hybridize to the reverse complementary sequence of the UR. The mediator is elongated if its 3′ end is bound to the UR sequence. Because of the elongation, a fluorescence signal is generated by either cleavage of the fluorophore and quencher or by unfolding of the hairpin structure of the UR. Case 1 shows the matching of the MP at the point mutation to be detected (green; G). The mediator is cleaved precisely after the position of the point mutation and thus the 3′ end of the mediator can be elongated at the UR leading to signal generation. In case 2, MP mismatch at the position of the targeted point mutation (orange; A) shifts the cleavage by exactly one nucleotide (purple; G). The UR is designed so that this nucleotide (nt) forms a so-called 1 nt flap, leading to an unbound 3′ end of the mediator when it hybridizes to the UR. This protruding nucleotide (G) prevents the mediator from being elongated and suppresses signal generation.

The principle of label-free MP with standardized UR, featuring target sequence independent signal optimization for qPCR, is described in more detail in Lehnert et al. [15]. Based on this concept, we demonstrate that digital MP PCR assays can also be developed without the need for tedious optimization of cycling parameters and nucleotide concentrations. Figure 1 shows the principle for the design of specific MP for point mutations. The reliable detection of point mutations needs specific probes to differentiate between a mismatch or a match at the position of the point mutation. During primer elongation, the mediator sequence is cleaved from the MP. It is assumed that the cleavage of the flap structure of the MP follows the mechanism described in 1993 by Lyamichev et al. [17]. The common nucleotide at the 3′ end of the generic mediator and the 5′ end of the target specific probe (purple G in Figure 1) is decisive for MP specificity as it is the complementary nucleotide to the point mutation. If the MP matches the point mutation (Figure 1, case 1), the mediator is cleaved precisely after the position of the point mutation. Subsequently, the cleaved mediator can hybridize to the UR and is elongated by the polymerase, and generates the fluorescence signal by either cleaving the fluorophore/quencher or unfolding the hairpin structure. The opposite case (no signal generation) occurs when the MP encounters a mismatch (other point mutation or wild type; Figure 1, case 2). Here, the common nucleotide cannot bind to the target sequence, and therefore the mediator is cleaved exactly after the position one nucleotide beyond the point mutation. The UR is designed in such a way that this additional nucleotide at the 3′ end of the mediator cannot bind to the UR sequence. This non-binding nucleotide generates a flap structure at the UR and prevents the elongation of the mediator at the UR sequence, so that the signal generation is suppressed. 

We recommend an assay design in which the position of the point mutation is located in the middle of the amplicon, thus primers need to be designed accordingly. This allows the MP to be placed on either of the two DNA strands (sense and antisense). This is necessary if the point mutation to be detected is a C or an A, in which case, the discriminating nucleotide of the MP (the 5′ end of the probe section) is a G or a T. This can lead to unspecific G/T pairings, for example, at the wild type or other point mutations, which can result in false positive PCR signals [23]. Therefore, if non-specific G/T bindings of the MP at multiple point mutations can occur, we recommend placing the MP on the opposite DNA strand (see Figure S3). In this case, the discriminating nucleotide at the mediator probe is a C or an A, which circumvents the G/T issue, resulting in a specific point mutation detection.

### 2.6. Assay Design

For assays applicable for liquid biopsy, it is important to design the amplicon length between 70 and 100 nucleotides. In 2011, Mouliere et al. [24] showed the fragmentation of ctDNA and concluded that amplicons smaller than 100 bp were necessary for liquid biopsy. Primers were designed using Primer-BLAST (parameter settings: amplicon maximum 100 nts, primer melting temperature: 60 °C, concentration of dNTPs of 0.8, concentration of divalent cations of 3.8) [25]. As previously demonstrated, the binding strength of MP at the target sequence and MP at UR has a major influence on the assay performance [19]. Thus, an optimized binding strength ∆G (at 60 °C) of approximately −20 kcal/mol for the MP at the target, and ∆G (at 60 °C) of approximately −11 kcal/mol of MP at UR was determined for MP PCR for point mutation detection. Gibb’s free energy ∆G was determined using Visual OMP (version 7.8.42.0, DNA Software, Plymouth, MA, USA) [26]. Visualization of oligonucleotides to avoid self-complementary or secondary structures was conducted using OligoPAD (version 0.3.9.4, GNWI mbH, Dortmund, Germany).

### 2.7. Setup of the DOE for MP ddPCR Assay Development

The MP ddPCR assay was developed using a DOE approach. As a model assay, we chose a 3-plex MP ddPCR assay for the detection of *KRAS* G12D, G12V, and wild type in the 3-plex Naica system (Stilla). *KRAS* G12D was analyzed in the red color channel with an Atto 647N fluorophore, *KRAS* G12V in the green color channel with a HEX fluorophore, and the wild type was detected in the blue color channel of the Naica device with a FAM fluorophore (for sequences see Supplemental Table S4). For statistical evaluation, the Minitab 19 software (Minitab, LLC) was used. The target value was the SNR, which was determined with the aim of maximizing its value through DOE, and a two-level factorial design was used. Before starting the DOE, the 3-plex MP ddPCR was performed with the following concentrations and conditions to evaluate the starting point before assay development. Reagent concentrations: primers 1 µM each, UR 250 nM each, MP 1 µM each, fluorescein 100 nM. Cycling conditions: hot start of 95 °C for 5 min, then 45 cycles of denaturation at 95 °C for 15 s, and annealing/ extension at 56 °C for 40 s. On this basis, the following six input factors were defined, while the other parameters, the hot start, denaturation step, and fluorescein concentration remained constant. The lowest and highest values of each factor tested in the DOE are given in parentheses:

- Annealing temperature (54 °C/60 °C)
- Annealing time (30 s/60 s)
- MP concentration (500 nM/1200 nM)
- MP:UR ratio (2:1/5:1)
- Forward primer (500 nM/1200 nM)
- Reverse primer (500 nM/1200 nM)

To reduce the number of experiments, a fractional factorial design was used instead of a full factorial design. In detail, a ½ fraction factorial design with resolution VI was chosen, resulting in 2^6–1^ = 32 experiments [27]. The SNR was calculated for each of the three-color channels (blue, green, red) for each experiment (see Table S5). The SNR was calculated by dividing the mean value of positive droplets by the mean value of negative droplets in the respective color channel.

## 3. Results and Discussion

### 3.1. MP ddPCR Assay Development Using DOE

We successfully demonstrated that the multiplex MP ddPCR could be developed and optimized using a DOE approach, leading to efficient use of resources by reducing the number of experiments to a statistically relevant level. The aim of the DOE was to increase the SNR in all three color channels (blue, green, red) of the ddPCR Naica system. This target value was chosen to improve droplet cluster separation between positive and negative droplets for a more sensitive quantification of point mutations. 

A triplex MP ddPCR for *KRAS* G12D, G12V, and wild type was performed as the starting point for the optimization of the SNR. However, if the threshold separating the positive and negative droplets cannot be clearly set, this may lead to a bias in the calculation of the target concentrations [28]. After performing the DOE, the SNR could be significantly increased in all three color channels (blue, green, red), see Figure S1. The best result for the sum of all three SNRs (see Table S5) was achieved with the following input factors:

- Annealing temperature = 54 °C
- Annealing time = 60 s
- MP concentration = 1200 nM
- MP:UR ratio = 5:1 (1200 nM MP and 240 nM UR)
- Forward primer = 500 nM
- Reverse primer = 1200 nM

With these parameters, the SNR could be increased from 1.8 to 2.0 in the blue channel, from 2.9 to 3.9 in the green channel, and from 6.4 to 7.3 in the red channel (compared to the values before DOE from the initial 3-plex MP PCR, see Figure S1). By applying this optimized parameter set, it is possible to clearly differentiate between positive and negative droplet clusters in all three color channels. It is worth noting that a distinct cluster separation is also achieved in the blue color channel with the basal fluorescence signal. This is essential for accurate and sensitive target quantification, especially at low target concentrations, as is the case in liquid biopsy. 

This DOE enabled the development of an optimized multiplex MP ddPCR assay for the detection of point mutations in liquid biopsy. It is intended to be used as a starting point for other point mutation panels without further optimization. The basis for this optimization-free assay is the fact that the MP PCR is a two-step process in which target detection takes place independently of signal generation [15,16]. This allows for the separate optimization of the signal generation and the transfer to an unlimited number of different targets. The use of an optimization-free MP ddPCR assay is a huge advantage over conventional dPCR systems, where assay concentrations and cycling conditions need to be optimized individually for each assay. Thus, the presented optimization-free MP ddPCR assay allows for flexible target panel compositions that can be easily adapted during patient-specific monitoring.

Analysis of the factorial design of the DOE in Minitab identified three effects of the input factors that have a significant influence on the 3-plex MP ddPCR assay, see Figure S2. The highest effect on the response (sum of SNR) comes from the two-factor interaction of the MP concentration and the MP:UR ratio, followed by the annealing time and the annealing temperature. This is related to the intrinsic two-step process of the MP PCR [16]. As we observed in the DOE, a high MP concentration (1200 nM), together with a high MP:UR ratio (5:1), a long annealing time (60 s) and a low annealing temperature (54 °C) led to the best result. This can be attributed to the fact that the MP needs to be available in a higher concentration compared to the limiting UR concentration so as to favor MP hybridization to the target instead of to the UR, leading to more effective cleavage and release of the mediator [29]. Due to the two separate detection and signal generation steps, the cleaved mediators need some time to diffuse to the UR molecules, thus a longer annealing time seems to be more favorable. A lower annealing temperature facilitates hybridization of the primers and MP to the target and of the cleaved mediator to the UR. However, too low temperatures impair specificity.

As optimized conditions in this DOE were identified at the limits of the selected design, it can be assumed that further optimization is possible. However, the achieved sensitivity and specificity already fulfills the clinical requirements for liquid biopsy.

### 3.2. Assay Characterization of 2-Plex and 4-Plex MP ddPCR Assay

After DOE assay development, the optimized parameters were fixed and applied for the assay characterization of the 2-plex and 4-plex MP ddPCR assays. The performance characteristics of the MP ddPCR and the reference assays are listed in Table 1.

We compared the performance of the novel 2-plex and 4-plex MP ddPCR assays with the 2-plex LNA ddPCR reference assay. Both MP PCR assay types showed comparable results with the LNA assay (LoB and LoD). The lowest measurable allele frequencies for the 2-plex MP ddPCR assay ranged from 0.004% to 0.050%. For the 4-plex MP ddPCR assays, they ranged from 0.009% to 0.383% and for the reference assays from 0.011% to 0.170%. This shows that all the assays have comparable sensitivities independent of the technology used. The data for the 2-plex MP ddPCR assay are shown in Tables S7–S13, the regression curves in Supplementary Figures S5–S11 and the data for the 4-plex MP ddPCR assay in Table S14 (calculated allele frequencies) and Tables S15 and S16 (copy numbers (LoB and LoD)). For the 2-plex and 4-plex MP ddPCR assays, the LoB was 0 copies/mL, except for *KRAS* G13D and *BRAF* V600E in the 4-plex approach. Here, the LoBs increased to 9.82 copies/mL and 16.29 copies/mL plasma, respectively. In these cases, the limit of quantification did not equal the limit of detection and was increased compared to a LoB of 0 copies/mL. The reason for this discrepancy in the case of panel 3 could be that the optimization of the assay conditions was conducted only in a *KRAS* 3-plex assay with just one wild type sequence and two mutant sequences and not with two wild type sequences, as was the case for panel 3. Furthermore, the amplicon of the *BRAF* target is AT rich, which results in longer primer and probe sequences to achieve comparable melting temperatures as for the *KRAS* targets. Thus, the *BRAF* amplicon had 133 bp compared to 98 bp for the *KRAS* panels.

The previously shown results are from spiking experiments with synthetic DNA fragments. The LoB was performed with eluates from plasma from healthy volunteers. The literature provides the average cfDNA concentration for healthy people as between 5 and 10 ng/mL plasma [30,31]. This results in a calculated cfDNA wild type concentration of 10 to 20 copies/µL in a reaction volume of 25 µL. The 2-plex MP ddPCR assay showed a wild type cfDNA concentration of 21.72 ± 4.69 copies/µL, which is in good accordance with the literature. This supports the thesis that the MP PCR technology can provide reliable measurements when using real CRC patient samples.

**Figure 2:**
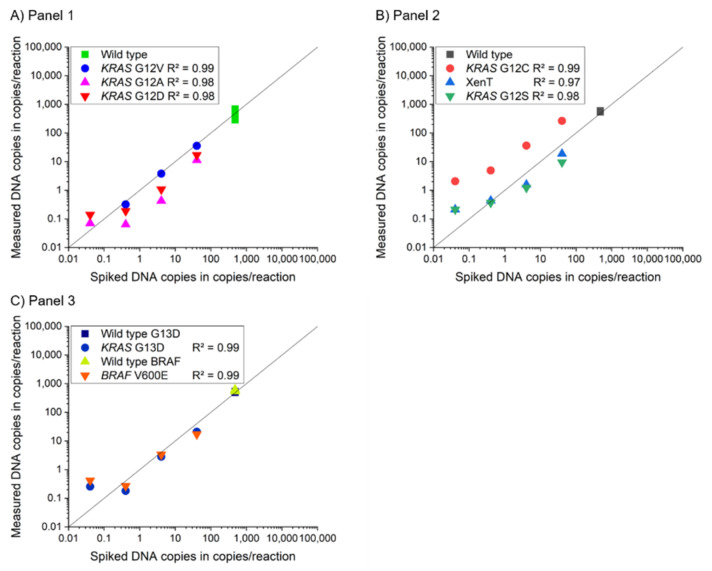
(**A**–**C**) Dilution series experiments for 4-plex MP ddPCR assays of panels 1 to 3. These assays were evaluated regarding sensitivity and linearity for each target. All assays showed a R^2^ ≥ 0.97 in the range from 1 to 100 DNA copies/reaction in the spiking experiment. The lowest tested concentration (0.01 DNA copies/reaction) was used for sensitivity testing and was not included in the evaluation of the determination coefficient as the influence of the subsampling effect dominates for these low concentrations. The deviation from the expected signal for the G12S target could result from pre-analytical preparation steps (e.g., for the stock solution).

The three 4-plex MP ddPCR assay panels all showed a R^2^ ≥ 0.98 for the mutant targets, which is comparable to the R^2^ of the 2-plex assays. Figure 2 shows the linearity and R^2^ of the 4-plex panels.

The LoB and AF results for the 4-plex MP ddPCR assays were slightly worse compared to the results for the 2-plex MP ddPCR assays. Nevertheless, with a 4-plex approach, twice as many markers in the same volume could be analyzed, saving patient samples and reagents. The optimized conditions identified in the DOE were at the limits of the selected experimental design. These were sufficient to develop an optimization-free 3-plex MP ddPCR assay that we have shown can be flexibly applied to different marker panels of point mutations in liquid biopsy. We show the capability of multiplexing with fixed oligonucleotide concentrations and cycling conditions independent of the targets and the respective point mutations, thus allowing optimization-free multiplexing with high specificity and sensitivity. Nevertheless, transferring this assay to other disease markers, sample types, or readout devices, or to other fluorescence/quencher types, might require a new experimental design. However, this new design could be determined analogously to the DOE presented here.

### 3.3. MP ddPCR Assay Validation with Clinical Samples

**Figure 3:**
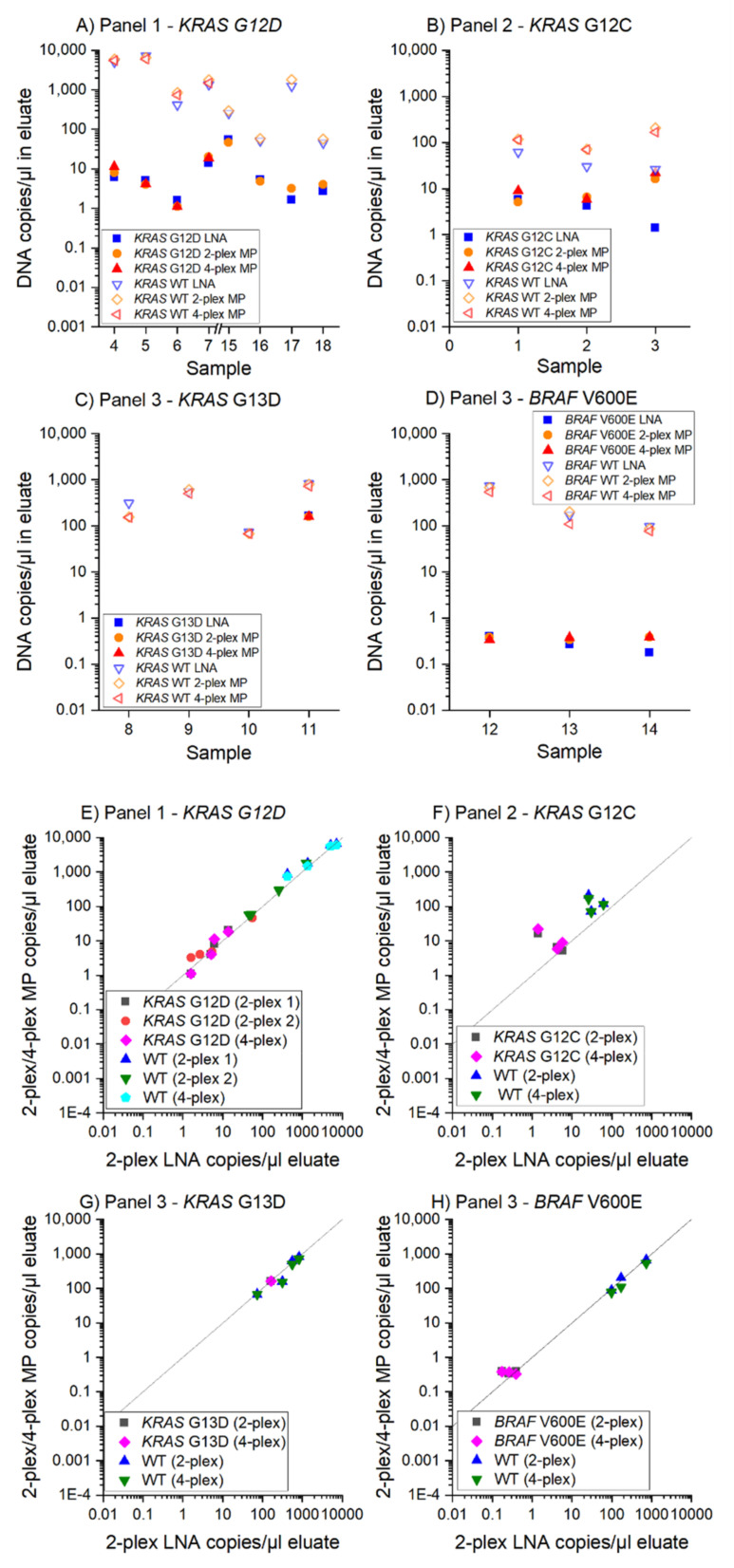
MP ddPCR assay validation with patient samples. The 2-plex and 4-plex MP ddPCR assays were compared to the 2-plex LNA reference assays. Wild type and mutant measurements are shown for four point mutations (*KRAS* G12D, G12C, G13D, and *BRAF* V600E) in 18 different patient samples. (**A**–**D**) DNA copies/µL in the eluate for reference and MP ddPCR assays, (**E**–**H**) Evaluation of 2-plex and 4-plex MP ddPCR and reference assay. The deviation of the data for *KRAS* G12C in (**B**,**F**) could result from the different measurement time points (LNA measurements in 2019, MP measurements in 2020).

The performance of the 2-plex and 4-plex MP ddPCR assays previously characterized by spiking experiments were validated with clinical samples. Figure 3 shows comparable results for the measurement of the clinical samples with both the 2-plex and 4-plex MP ddPCR assays.

Our MP ddPCR assays were able to assign 100% of the previously tested samples (LNA reference assay) as correctly positive or correctly negative (Figure 3A–D) with a high concordance between the 2-plex MP ddPCR assay and the 2-plex LNA assay (Figure 3E,F):

- 9/9 samples *KRAS* G12D positive
- 3/3 samples *KRAS* G12C positive
- 1/1 sample *KRAS* G13D positive
- 2/2 samples *KRAS* G13D negative and
- 3/3 samples *BRAF* V600E positive

**Figure 4:**
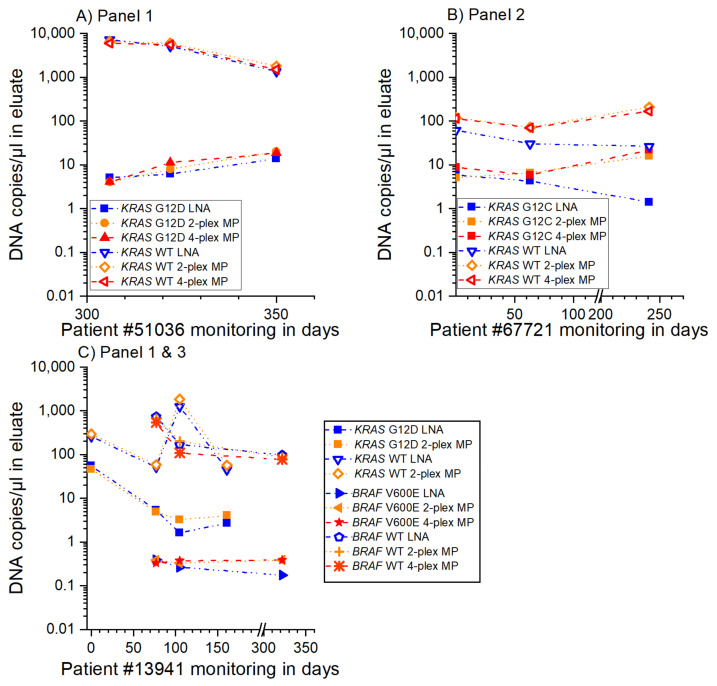
Therapy monitoring of patient samples for different sampling time points. The 2-plex and 4-plex MP ddPCR assay results coincided with the LNA reference assay results. (**A**–**C**) Therapy monitoring of three patients with three to five sampling time points and measurement of the three cancer-associated point mutations *KRAS* G12D, G12C, and *BRAF* V600E.

Therapy monitoring for three patients with three to five sampling time points within one year is shown in Figure 4. The graphs show the applicability of the presented 2-plex and 4-plex MP ddPCR assays for clinical applications. All assays provide reliable information on the increase or decrease in the level of cfDNA in both the mutant and wild type sequences. The comparison between the 2-plex and 4-plex MP ddPCR assays and the 2-plex LNA reference assay proves the applicability of optimization-free multiplex MP ddPCR assays as well as of the applied assay design guidelines. These include the positioning of the point mutation in the middle of the amplicon so that the MP can be designed on the sense or the antisense strand. This design is used especially for the G/T mismatch binding in the case of (e.g., *KRAS* G12V). Here, the MP for the wild type could bind to the position of the point mutation and thus result in an unspecific signal. To prevent this, mediator probes for the wild type sequences that have a G at the position of the point mutation were designed on the antisense strand. This resulted in a high specificity, as shown in Figure S4. The concept of the 1 nt flap at the UR in the case of a mismatch was also proven with these experiments.

## 4. Conclusions

This work shows specific and sensitive 2-plex and 4-plex MP ddPCR assays and their application in the monitoring of point mutations in liquid biopsy samples. Moreover, the performance of the presented 2-plex and 4-plex MP ddPCR assays is comparable to previously published 2-plex LNA reference assays. We provide exemplarily application guidelines for the MP ddPCR assay design for point mutations in liquid biopsy. With fixed PCR conditions for all presented MP ddPCR assays for ctDNA, we demonstrated an optimization-free multiplex assay in a clinical setting. With the presented strategy, fast and robust assay design is possible for patient individual panels. The necessity of only one cycling condition for all assays is not only a prerequisite for multiplexing, but is also important for certification aspects. The ease of creating multiplex panels allows for fast and flexible panel adaptions depending on patient treatment and therapy response. This makes multiplex MP ddPCR assays a promising tool for guiding modern personalized therapies in cancer. In the future, we aim to apply our guidelines for cancer entities other than CRC. Our vision is to create a multiplex assay platform for ctDNA-based liquid biopsy, which is easy to tailor to individual treatment strategies and allows for efficient routine diagnosis and improved treatment response monitoring.

## Figures and Tables

**Table 1:** Characterization of 2-plex and 4-plex MP ddPCR assays (LoB, LoD, and R^2^) on Stilla Naica system. Comparison to reference LNA assays on the Bio-Rad device.

Panel	Gene	Mutation	Change of Nucleotide	Change of Amino Acid	2-Plex Mediator Probe ddPCR			4-Plex Mediator Probe ddPCR			2-Plex Reference Assay		
					LoB (Copies/mL Plasma)	LoD (% AF)	R^2^ 2-Plex	LoB (Copies/mL Plasma)	LoD (% AF)	R^2^ 4-Plex	LoB (Copies/mL Plasma)	LoD (% AF)	R^2^ 2-Plex
1	*KRAS*	Codon 12	WT										
	*KRAS*	G12D	c.35G>A	p.Gly12Asp	0.00	0.008	0.99	0.00	0.024	0.98	3.26	0.032	0.99
	*KRAS*	G12A	c.35G>C	p.Gly12Ala	0.00	0.004	0.99	0.00	0.009	0.98	0.00	0.074	0.99
	*KRAS*	G12V	c.35G>T	p.Gly12Val	0.00	0.011	0.99	0.00	0.047	0.99	2.21	0.011	0.99
2	*KRAS*	Codon 12	WT										
	*KRAS*	G12S	c.34G>A	p.Gly12Ser	0.00	0.019	0.99	0.00	0.040	0.98	1.43	0.097	0.99
	*KRAS*	G12C	c.34G>T	p.Gly12Cys	0.00	0.015	0.99	0.00	0.383	0.99	0.00	0.067	0.99
	*Xenopus tropicalis (XenT)* extraction control												
3	*KRAS*	Codon 13	WT										
	*KRAS*	G13D	c.38G>A	p.Gly13Asp	0.00	0.050	0.99	9.82	0.034	0.99	1.68	0.170	0.99
	*BRAF*	Codon 600	WT										
	*BRAF*	V600E	c.1799T>A	p.Val600Glu	0.00	0.039	0.99	16.29	0.050	0.99	5.90	0.047	0.99

## Data Availability

The data source presented in this study are available on request from the corresponding authors.

## Supplementary Materials

The following are available online at https://www.mdpi.com/article/10.3390/cancers13225742/s1, Table S1: 2-plex MP ddPCR assays, Table S2: 4-plex MP ddPCR panels, Table S3: Sequences of the primers, mediator probes, and universal reporters for the 2-plex MP and 4-plex MP panels, Table S4: Sequences of the primers, mediator probes, and universal reporters for the 3-plex MP ddPCR detecting *KRAS* G12D, G12V and wild type for DOE, Table S5: Design of the 32 experiments from the DOE of the 3-plex MP ddPCR including the input factors and the resulting signal-to-noise ratios of each color channel and the sum of all signal-to-noise ratios, Table S6: Cycling parameters for LNA reference assay in Bio-Rad system, Table S7: LoB and LoD data for 2-plex MP ddPCR assay characterization of *KRAS* G12A and its corresponding wild type in Stilla system, Table S8: LoB and LoD data for 2-plex MP ddPCR assay characterization of *KRAS* G12V and its corresponding wild type in Stilla system, Table S9: LoB and LoD data for 2-plex MP ddPCR assay characterization of *KRAS* G12D and its corresponding wild type in Stilla system, Table S10: LoB and LoD data for 2-plex MP ddPCR assay characterization of *KRAS* G12S and its corresponding wild type in Stilla system, Table S11: LoB and LoD data for 2-plex MP ddPCR assay characterization of G12C and its corresponding wild type in Stilla system, Table S12: LoB and LoD data for 2-plex MP ddPCR assay characterization of *KRAS* G13D and its corresponding wild type in Stilla system, Table S13: LoB and LoD data for 2-plex MP ddPCR assay characterization of *BRAF* V600E and its corresponding wild type in Stilla system, Table S14: LoB and LoD data for 4-plex MP ddPCR assay characterization with LionheartLX, Table S15: LoD copies/reaction data for 4-plex MP ddPCR assay characterization with LionheartLX, Table S16: LoB copies/reaction data for 4-plex MP ddPCR assay characterization with LionheartLX, Table S17: Data for measurements of patient samples with 2-plex LNA, 2-plex MP ddPCR, and 4-plex MP ddPCR assays with Biorad (2-plex LNA), Stilla Naica (2-plex MP ddPCR) and LionheartLX (4-plex MP ddPCR). Figure S1: 3-D scatterplots (generated by the Stilla CrystalMiner software) of the 3-plex MP ddPCR for *KRAS* G12D/G12V and wild type, Figure S2: Pareto chart of the effects of the 3-plex MP ddPCR as a result of the factorial design analysis of the DOE (generated with Minitab), Figure S3: Recommended MP PCR assay design for point mutations: If the point mutation to be detected is a C or an A, the discriminating nucleotide at the mediator probe (5′ end of the probe section) is a G or a T, Figure S4: Specificity tests of mediator probes in a qPCR device (RotorgeneQ, Qiagen) in singleplex reactions, Figure S5: LoB and LoD results with linear fit for 2-plex MP ddPCR assay characterization of *KRAS* G12A and its corresponding wild type in Stilla system, Figure S6: LoB and LoD results with linear fit for 2-plex MP ddPCR assay characterization of *KRAS* G12V and its corresponding wild type in Stilla system, Figure S7: LoB and LoD results with linear fit for 2-plex MP ddPCR assay characterization of *KRAS* G12D and its corresponding wild type in Stilla system, Figure S8: LoB and LoD results with linear fit for 2-plex MP ddPCR assay characterization of *KRAS* G12S and its corresponding wild type in Stilla system, Figure S9: LoB and LoD results with linear fit for 2-plex MP ddPCR assay characterization of *KRAS* G12C and its corresponding wild type in Stilla system, Figure S10: LoB and LoD results with linear fit for 2-plex MP ddPCR assay characterization of *KRAS* G13D and its corresponding wild type in Stilla system, Figure S11: LoB and LoD results with linear fit for 2-plex MP ddPCR assay characterization of *BRAF* V600E and its corresponding wild type in Stilla system.
