# Supplementary material for: Stringent Base Specific and Optimization-Free Multiplex Mediator Probe ddPCR for the Quantification of Point Mutations in Circulating Tumor DNA
Source: Cancers (Basel). 2021 Nov 16;13(22):5742. doi: 10.3390/cancers13225742 (PMC8616434; doi:10.3390/cancers13225742)
Supplement: Supplementary file 1 [file cancers-13-05742-s001.zip › cancers-1423472-supplementary.pdf]

Supplemental Data

Stringent base specific and optimization-free multiplex mediator probe ddPCR for the quantification of point mutations in circulating tumor DNA

Franziska Schlenker[1†], Elena Kipf[1†], Max Deuter[2], Inga Höffkes[1], Michael Lehnert[1], Roland Zengerle[1,3], Felix von Stetten[1,3], Florian Scherer[2], Julius Wehrle[2], Nikolas von Bubnoff[2,4], Peter Juelg[1], Tobias Hutzenlaub[1,3 ‡] and Nadine Borst[1,3, *, ‡]

[1] Hahn-Schickard, Georges-Koehler-Allee 103, 79110 Freiburg, Germany

[2] Department of Medicine I, Medical Center – University of Freiburg, Faculty of Medicine, University of Freiburg, Freiburg, Germany

[3] Laboratory for MEMS Applications, IMTEK - Department of Microsystems Engineering, University of Freiburg, Georges-Koehler-Allee 103, 79110 Freiburg, Germany

[4] Department of Hematology and Oncology, University Hospital Schleswig-Holstein, Campus Lübeck, Germany

* Corresponding author

† contributed equally, ‡ contributed equally

Content

1. Sequences and Oligo information 3
DNA accession number: 3
2. DOE data 7
3. Assay design 11
4. Cycling Parameters LNA assay 12
5. MP PCR assay data 13
5.1. Singleplex MP PCR specificity tests in qPCR device 13
5.2. 2-plex MP ddPCR assay LoB and LoD data in Stilla dPCR device 14
5.3. 4-plex MP ddPCR assay LoB and LoD data 21
5.4. MP ddPCR assay performance data with patient samples 23
References 24

1. Sequences and Oligo information

Supplemental table S 1 *2-plex MP ddPCR assays. Each 2-plex MP ddPCR assay is able to detect one mutation and its respective wild type sequence. List of mutations, nucleotide sequence in area of interest (point mutation), change of nucleotide, change of amino acid, rs number, chromosome and position.*

2-plex MP ddPCR assays

	Gen	Mutation	Codon	5' - end										3'- end	Change of nucleotide	Change of amino acid	rs number (ncbi)	Chromosome and position
1	*KRAS*	WT Codon 12/13	12	...	G	C	T	G	G	T	G	G	C	...	WT			
	KRAS	G12D	12	...	G	C	T	G	A	T	G	G	C	...	c.35G>A	p.Gly12Asp	rs121913529	GRch38 Chr12:25245350
2	*KRAS*	WT Codon 12/13	12	...	G	C	T	G	G	T	G	G	C	...	WT			
	KRAS	G12A	12	...	G	C	T	G	C	T	G	G	C	...	c.35G>C	p.Gly12Ala	rs121913529	GRch38 Chr12:25245350
3	*KRAS*	WT Codon 12/13	12	...	G	C	T	G	G	T	G	G	C	...	WT			
	KRAS	G12V	12	...	G	C	T	G	T	T	G	G	C	...	c.35G>T	p.Gly12Val	rs121913529	GRch38 Chr12:25245350
4	*KRAS*	WT Codon 12/13	12	...	G	C	T	G	G	T	G	G	C	...	WT			
	KRAS	G12S	12	...	G	C	T	A	G	T	G	G	C	...	c.34G>A	p.Gly12Ser	rs121913530	GRch38 Chr12:25245351
5	*KRAS*	WT Codon 12/13	12	...	G	C	T	G	G	T	G	G	C	...	WT			
	KRAS	G12C	12	...	G	C	T	T	G	T	G	G	C	...	c.34G>T	p.Gly12Cys	rs121913530	GRch38 Chr12:25245351
6	*KRAS*	WT Codon 12/13	12	...	G	C	T	G	G	T	G	G	C	...	WT			
	KRAS	G13D	13	...	G	C	T	T	G	T	G	A	C	...	c.38G>A	p.Gly13Asp	rs112445441	GRch38 Chr12:25245347
7	*BRAF*	WT	600		T	T	T	C	A	C	T	G	T		WT			
	BRAF	V600E	600		T	T	T	C	T	C	T	G	T		c.1799T>A	p.Val600Glu	rs113488022	GRch38 Chr7:140753336

Supplemental table S 2 *4-plex MP ddPCR panels. Each panel is able to detect up to three mutations and their respective wild type sequence. List of mutations, nucleotide sequence in area of interest (point mutation), change of nucleotide, change of amino acid, rs number, chromosome and position. Panel 2 is used for the detection of KRAS G12S, G12C, their wild type and an extraction control (Xenopus tropicalis).*

4-plex MP ddPCR panels

Panel	Gen	Mutation	Codon	5' - end										3'- end	Change of nucleotide	Change of amino acid	rs number (ncbi)	Chromosome and position
1	*KRAS*	WT Codon 12/13	12	...	G	C	T	G	G	T	G	G	C	...	WT			
	KRAS	G12D	12	...	G	C	T	G	A	T	G	G	C	...	c.35G>A	p.Gly12Asp	rs121913529	GRch38 Chr12:25245350
	KRAS	G12A	12	...	G	C	T	G	C	T	G	G	C	...	c.35G>C	p.Gly12Ala	rs121913529	GRch38 Chr12:25245350
	KRAS	G12V	12	...	G	C	T	G	T	T	G	G	C	...	c.35G>T	p.Gly12Val	rs121913529	GRch38 Chr12:25245350
2	*KRAS*	WT Codon 12/13	12	...	G	C	T	G	G	T	G	G	C	...	WT			
	KRAS	G12S	12	...	G	C	T	A	G	T	G	G	C	...	c.34G>A	p.Gly12Ser	rs121913530	GRch38 Chr12:25245351
	KRAS	G12C	12	...	G	C	T	T	G	T	G	G	C	...	c.34G>T	p.Gly12Cys	rs121913530	GRch38 Chr12:25245351
	Xenopus Tropicalis (XenT) extraction control																	
3	*KRAS*	WT Codon 12/13	12	...	G	C	T	G	G	T	G	G	C	...	WT			
	KRAS	G13D	13	...	G	C	T	T	G	T	G	A	C	...	c.38G>A	p.Gly13Asp	rs112445441	GRch38 Chr12:25245347
	BRAF	WT	600		T	T	T	C	A	C	T	G	T		WT			
	BRAF	V600E	600		T	T	T	C	T	C	T	G	T		c.1799T>A	p.Val600Glu	rs113488022	GRch38 Chr7:140753336

DNA accession number:

- ***KRAS* WT, G12D, G12V, G12A, G12S, G12C and G13D: N_000012.12**
- ***BRAF* WT and V600E, G12V, G12A, G12S, G12C and G13D: N_000007.14**

Supplemental table S 3 *Sequences of the primers, mediator probes and universal reporters for the 2-plex MP and 4-plex MP panels.*

Name	*Sequence 5'-3'*	*5' mod*	*3' mod*	*Internal*
Fwd_Primer_KRAS (1)	GGCCTGCTGAAAATGACT	-	-	-
Rev_Primer_KRAS (1)	ACAAAATGATTCTGAATTAGCTGTA	-	-	-
MP_UR05_G12D (1)	ATGTCCCAGGTGCATGGCGTAGGCAAGAGTGCCTTGACGAT	-	C3 Spacer	-
UR05_red	GACCGGCTAAGACGCGCCGGT7TGT*TGCACCTGGGACAT*CGACTAT	BHQ-2	C3 Spacer	7=dC-Atto 647N
MP_UR02_WT	CTCCAGTTCGGTCCAGCTCCAACTACCACAAGTTTATATTCAG	-	C3 Spacer	-
UR02_green	ATTGCGGGAGATGAGACCCGCAA8TGTTCACT*GACCGAACTGGAG*CA	BMN-Q535	C3 Spacer	8=dT-FAM
MP_UR06_G12V (1)	AGGTAGGCTCACTTGGCGTAGGCAAGAGTGCCTTGACGAT	-	C3 Spacer	-
UR06_yellow	GACGCGTAGTACAGAACGCGT7TGTTC*AGTGAGCCTACCT*GCCTTC	BMN-Q535	C3 Spacer	7=dC-HEX
MP G12A Panel 1	GATACAGGGTCCACTGGCGTAGGCAAGAGTGCCTTGACG	-	C3 Spacer	-
MP G12V	GTAGGCTCACTGAACACAGCTCCAACTACCACAAGTTTATATTCAG	-	C3 Spacer	-
Xent Fwd. Primer	ACAATCTGGAGGGGCAAAGG	-	-	-
Xent Rev. Primer	CTCCACCTTCCAGCCATTGT	-	-	-
MP XenT	CTCGATACAGGGTCCAAGACAGGGATGATAAGTCTGGGGCAGC	-	C3 Spacer	-
MP WT- KRAS Panel 2	GTTCTGGGCTCTACAGCTCCAACTACCACAAGTTTATATTCAGTC	-	C3 Spacer	-
MP G12S	ATGTCCCAGGTGCAGTGGCGTAGGCAAGAGTGCCTTGACG	-	C3 Spacer	-
MP G12C	GCAGGTAGGCTGTGGCGTAGGCAAGAGTGCCTTGACG	-	C3 Spacer	-
MP WT- KRAS Panel 3	TCGATACAGGGTCCCACCAGCTCCAACTACCACAAGTTTATATTCAG	-	C3 Spacer	-

MP G13D	GGGCTCTACGACCACGTAGGCAAGAGTGCCTTGACGATAC	-	C3 Spacer	-
BRAF Fwd. Primer	GACCCACTCCATCGAGATTTC	-	-	-
BRAF Rev. Primer	GCTTGCTCTGATAGGAAAATGAG	-	-	-
MP BRAF WT	GGTAGGCTCACTGACTGTAGCTAGACCAAAATCACCTATTTTTACTGTGAG	-	C3 Spacer	-
MP BRAF V600E	CGATGTCCCAGGTCTGTAGCTAGACCAAAATCACCTATTTTTACTG	-	C3 Spacer	-
UR02_green	ATTGCGGGAGATGAGACCCGCAA8TGTTCACTGACCGAACTGGAGCA	BMN-Q-535	C3 Spacer	8 = dT-FAM
UR04_red	GACCGCACTAGTAGATGCGGT7TGTCGTGGACCCTGTATCGAGCA	BHQ2	C3 Spacer	7 = dC-Atto-647-N
UR06_red	GACGCGTAGTACAGAACGCGT7TGTTCAGTGAGCCTACCTGCCTTC	BHQ2	C3 Spacer	7 = dC-Atto-647-N
UR06_yellow	GACGCGTAGTACAGAACGCGT7TGTTCAGTGAGCCTACCTGCCTTC	BMN-Q-535	C3 Spacer	7=dCHEX
UR05_red	GACCGGCTAAGACGCGCCGGT7TGTTGCACCTGGGACATCGACTAT	BHQ2	C3 Spacer	7=dC-Atto647N
UR01_green	ATTGCGGGAGATGAGACCCGCAA8TGTTGGTCGTAGAGCCCAGAACGA	BMN-Q-535	C3 Spacer	8=dTFAM
UR04_yellow	GACCGCACTAGTAGATGCGGT7TGTCGTGGACCCTGTATCGAGCA	BMN-Q-535	C3 Spacer	7=dCHEX
UR01_red	GACCGGCCAAGACGCGCCGGT7TGTTGGTCGTAGAGCCCAGAACGA	BHQ2	C3 Spacer	7=dC-Atto647N
UR06_green	ATTGCGGGAGATGAGACCCGCAA8TGTTCAGTGAGCCTACCTGCCTTC	BMN-Q-535	C3 Spacer	8=dTFAM
UR04_orange	GACCGCACTAGTAGATGCGGT5TGTCGTGGACCCTGTATCGAGCA	BMN-Q-535	C3 Spacer	5=dC-Atto Rho 101
UR02_green	ATTGCGGGAGATGAGACCCGCAA8TGTTCACTGACCGAACTGGAGCA	BMN-Q-535	C3 Spacer	8 = dT-FAM
UR04_red	GACCGCACTAGTAGATGCGGT7TGTCGTGGACCCTGTATCGAGCA	BHQ2	C3 Spacer	7 = dC-Atto-647-N

UR06_red	GACGCGTAGTACAGAACGCGT7TGTTCAGTGAGCCTACCTGCCTTC	BHQ2	C3 Spacer	7 = dC-Atto-647-N
UR06_yellow	GACGCGTAGTACAGAACGCGT7TGTTCAGTGAGCCTACCTGCCTTC	BMN-Q-535	C3 Spacer	7=dCHEX

2. DOE data

Supplemental table S 4 *Sequences of the primers, mediator probes and universal reporters for the 3-plex MP ddPCR detecting KRAS G12D, G12V and wild type for DOE.*

Name	*Sequence*	*5' mod*	*3' mod*	*Internal*
Fwd_Primer_KRAS (1)	5'-GGCCTGCTGAAAATGACT-3'	-	-	-
Rev_Primer_KRAS (1)	5'-ACAAAATGATTCTGAATTAGCTGTA-3'	-	-	-
MP_UR05_G12D (1)	5'-atgtcccaggtgc**a**TGGCGTAGGCAAGAGTGCCTTGACGAT-3'	-	C3 Spacer	-
UR05_red	5'-GACCGGCTAAGACGCGCCGGT7TGT*tgcacctgggacat*CGACTAT-3'	BHQ-2	C3 Spacer	7=dC-Atto 647N
MP_UR02_WT	5'-ctccagttcggt**c**CAGCTCCAACTACCACAAGTTTATATTCAG-3'	-	C3 Spacer	-
UR02_green	5'-ATTGCGGGAGATGAGACCCGCAA8TGTTCACT*gaccgaactggag*CA-3'	BMN-Q535	C3 Spacer	8=dT-FAM
MP_UR06_G12V (1)	5'-aggtaggctcac**t**TGGCGTAGGCAAGAGTGCCTTGACGAT-3'	-	C3 Spacer	-
UR06_yellow	5'-GACGCGTAGTACAGAACGCGT7TGTTC*agtgagcctacct*GCCTTC-3'	BMN-Q535	C3 Spacer	7=dC-HEX

Small letters: Mediator section of the mediator probe; Underlined: Target-specific probe section of the mediator probe; Red and bold: Nucleotide at the mediator probe that discriminates the point mutation; Small italic letters: Binding position of the mediator at the universal reporter; UR0x: Universal reporter 0x; MP_UR0x: Mediator probe that activates universal reporter 0x.

Supplemental table S 5 *Design of the 32 experiments from the DOE of the 3-plex MP ddPCR including the input factors and the resulting signal-to-noise ratios of each color channel and the sum of all signal-to-noise ratios. A color scale displays the performance from green: good to red: poor.*

Input factors						Target value			**Sum signal-to-noise ratios**
Annealing temperature in °C	Annealing time in s	MP concentration in nM	MP/UR ratio	Fwd primer in nM	Rev primer in nM	Signal-to-noise ratio blue channel	Signal-to-noise ratio green channel	Signal-to-noise ratio red channel	
54	30	500	2:1	500	500	1.6	2.9	6.7	11.1
54	30	1200	2:1	500	1200	1.9	2.1	5.4	9.4
54	30	500	5:1	500	1200	1.4	3.7	5.2	10.3
54	30	1200	5:1	500	500	1.6	3.1	6.2	10.9
54	30	500	2:1	1200	1200	1.6	3.6	6.4	11.6
54	30	1200	2:1	1200	500	1.7	2.2	5.2	9.0
54	30	500	5:1	1200	500	1.3	3.4	5.4	10.1
54	30	1200	5:1	1200	1200	1.7	3.7	5.9	11.2
54	60	500	2:1	500	1200	1.9	3.4	7.2	12.6
54	60	1200	2:1	500	500	1.9	2.3	4.2	8.4
54	60	500	5:1	500	500	1.4	3.6	7.0	12.0
**54**	**60**	**1200**	**5:1**	**500**	**1200**	**2.0**	**3.9**	**7.3**	**13.1**
54	60	500	2:1	1200	500	1.8	2.8	7.3	11.8
54	60	1200	2:1	1200	1200	2.1	1.9	5.3	9.3
54	60	500	5:1	1200	1200	1.6	3.9	6.9	12.4
54	60	1200	5:1	1200	500	2.0	3.0	6.5	11.5
60	30	500	2:1	500	1200	1.5	2.2	5.3	9.0
60	30	1200	2:1	500	500	1.6	1.7	3.5	6.8
60	30	500	5:1	500	500	1.3	2.2	5.5	9.1
60	30	1200	5:1	500	1200	1.6	2.2	5.2	9.0
60	30	500	2:1	1200	500	1.5	2.4	5.8	9.7
60	30	1200	2:1	1200	1200	1.8	2.2	5.4	9.4
60	30	500	5:1	1200	1200	1.3	2.1	3.3	6.7
60	30	1200	5:1	1200	500	1.6	2.6	5.7	9.9
60	60	500	2:1	500	500	1.6	2.4	7.6	11.6
60	60	1200	2:1	500	1200	1.9	2.1	5.8	9.8
60	60	500	5:1	500	1200	1.4	3.2	5.9	10.4
60	60	1200	5:1	500	500	1.7	2.8	7.1	11.5
60	60	500	2:1	1200	1200	1.6	2.5	6.9	11.0
60	60	1200	2:1	1200	500	1.8	2.0	5.6	9.4
60	60	500	5:1	1200	500	1.3	2.9	5.1	9.2
60	60	1200	5:1	1200	1200	1.6	2.6	7.0	11.2

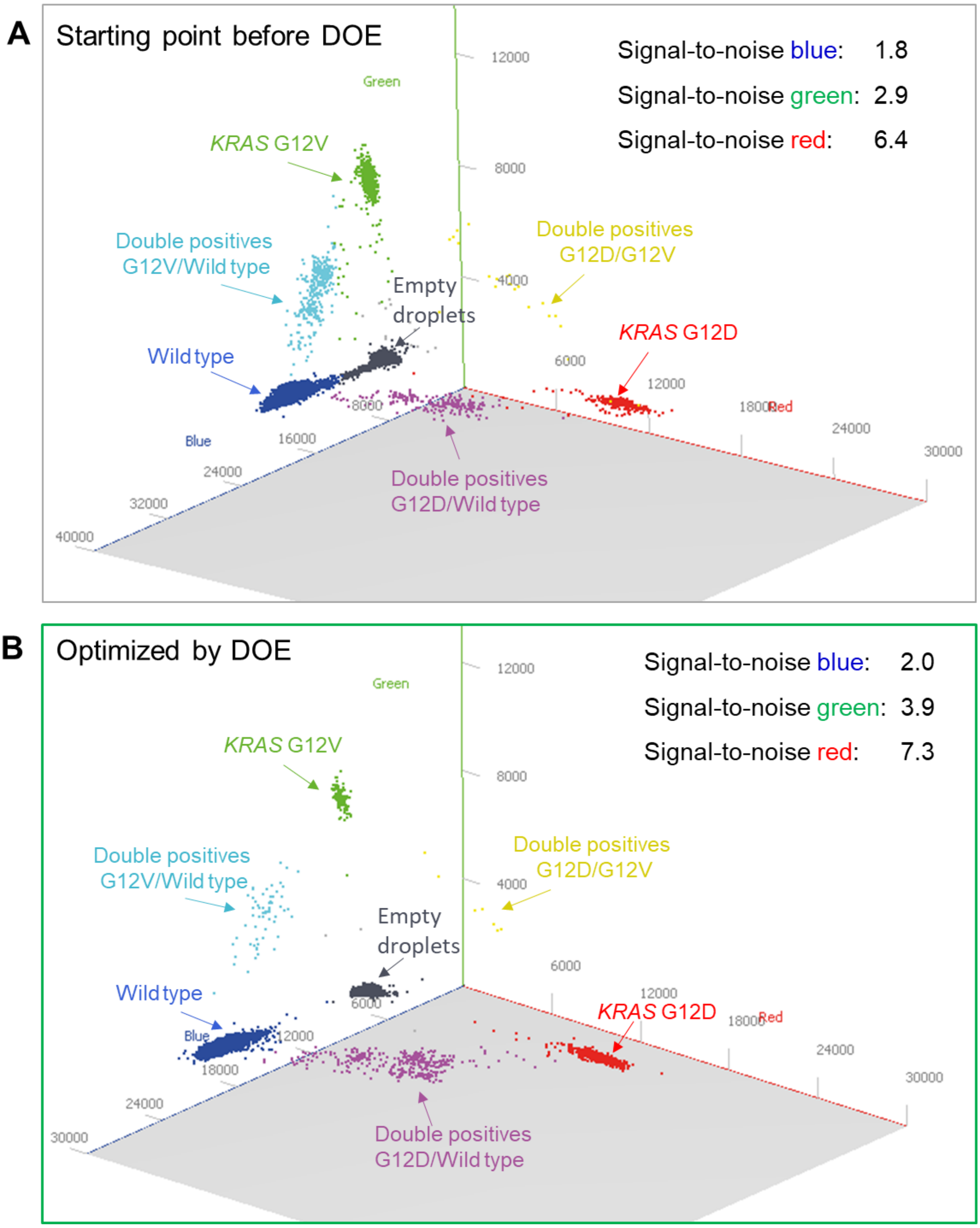

Supplemental figure S 1 *3-D scatterplots (generated by the Stilla CrystalMiner software) of the 3-plex MP ddPCR for KRAS G12D/ G12V and wild type.* ***A)*** *Starting point of the 3-plex MP before DOE, together with the calculated signal-to-noise ratios for all three color channels. Therein, especially in the blue color channel detecting the wild type, the droplet clusters of positive and negative droplets could hardly be distinguished. This is due to the fact that fluorescein also provides a basal fluorescence signal in the blue channel, which is required for droplet detection by the CrystalMiner software.* ***B)*** *Optimized 3-plex MP ddPCR after DOE (annealing temp.: 54 °C, annealing time: 60 s, MP conc.: 1200 nM, MP/ UR ratio: 5:1; fwd primer: 500 nM, rev primer: 1200 nM), and the corresponding signal-to-noise ratios for each color channel.*

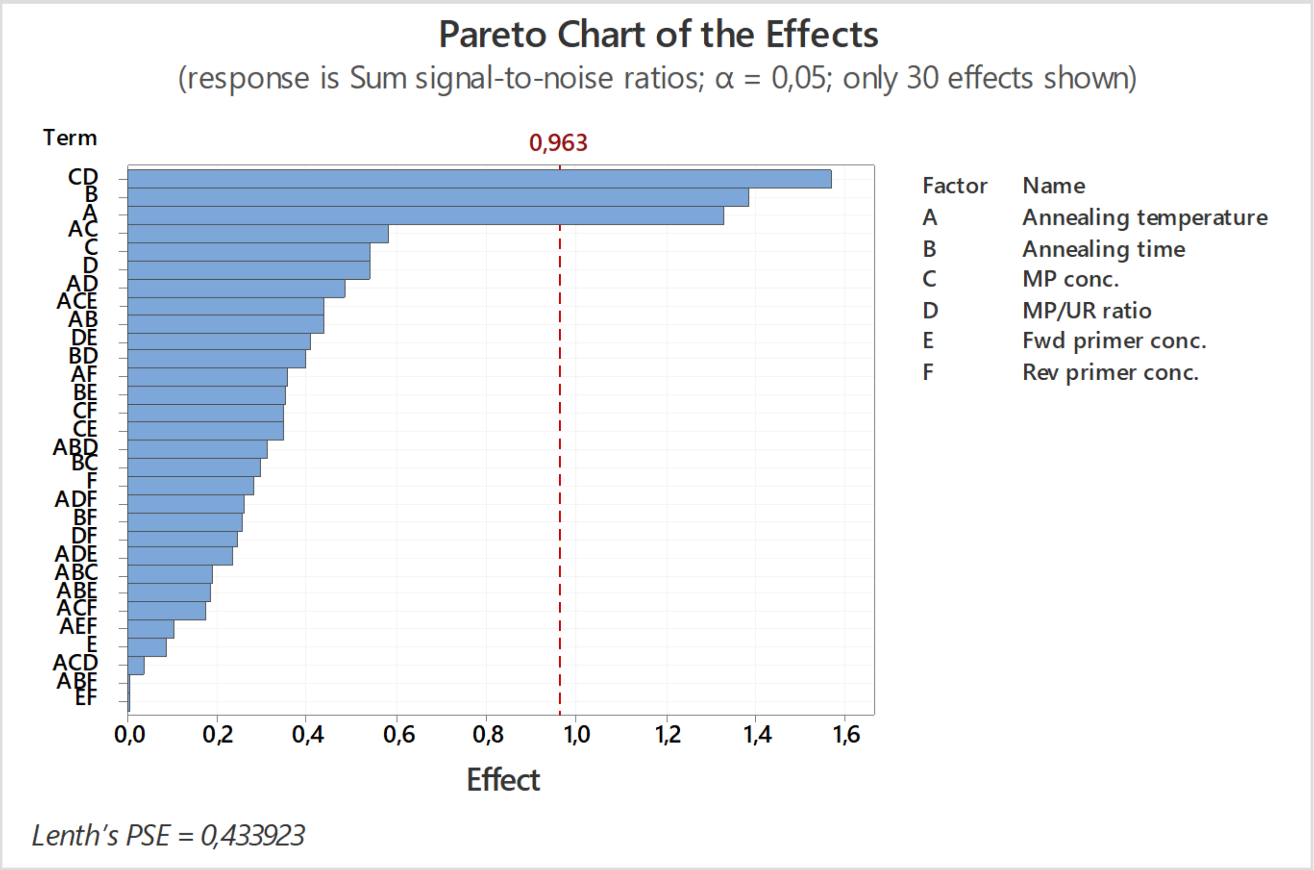

Supplemental figure S 2 *Pareto chart of the effects of the 3-plex MP ddPCR as a result of the factorial design analysis of the DOE (generated with Minitab). A Pareto chart shows the values of the effects from the largest to the smallest effect. Bars that cross the red reference line are statistically significant. Here, the two-factor interaction of the MP concentration and MP/UR ratio, the annealing time, and the annealing temperature have significant effects on the response sum of all three signal-to-noise ratios (blue, green, red).*

3. Assay design

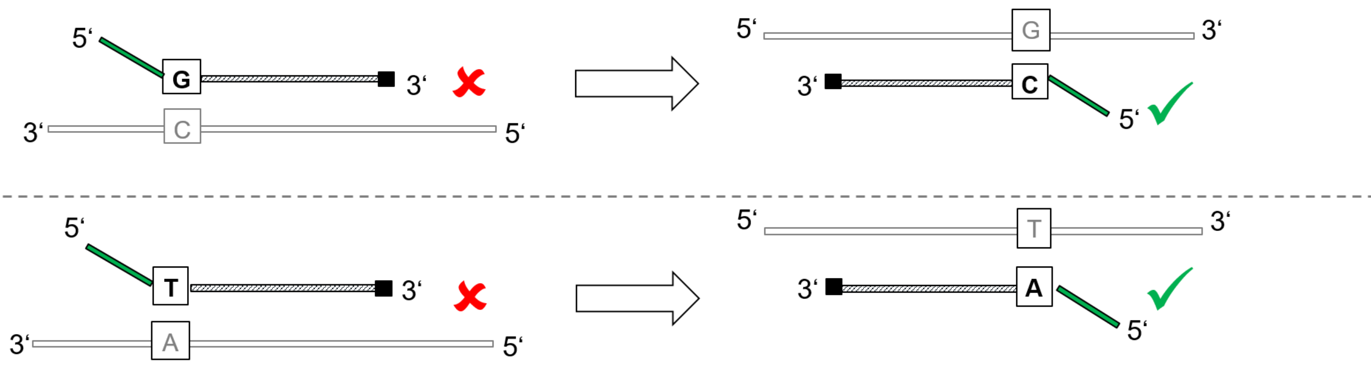

Supplemental figure S 3 *Recommended MP PCR assay design for point mutations: If the point mutation to be detected is a C or an A, the discriminating nucleotide at the mediator probe (5’ end of the probe section) is a G or a T. This can result in unspecific G/T paring with other markers or the wild type leading to false positive signals. Thus, we recommend to place the mediator probe on the opposite DNA strand if there is a risk of non-specific binding to other targets. This leads to mediator probes with the discriminating nucleotide C or A, resulting in specific point mutation detection. To allow placement of the mediator probe on both DNA strands, it is necessary to place the point mutation in the middle of the amplicon and to design the primers accordingly.*

4. Cycling Parameters LNA assay

Supplemental table S 6 *Cycling parameters for LNA reference assay in Bio-Rad system.*

Cycling Step	Temperature, °C	Time	Number of Cycles
**Enzyme activation**	95	10 min	-
**Denaturation**	95	30 sec	50x<br>(KRAS G13D, G12S, G12A, G12C, G12D, G12R,)<br>40x<br>(KRAS G12V)
**Annealing/ Extension**	62.0 (KRAS G13D, G12S)<br>59.4 (KRAS G12V, G12C, G12A)<br>55 (KRAS G12D, G12R)	90 sec	
**Enzyme inactivation**	98	10 min	-
**Hold (optional)**	12	Infinite	-

5. MP PCR assay data

5.1. Singleplex MP PCR specificity tests in qPCR device

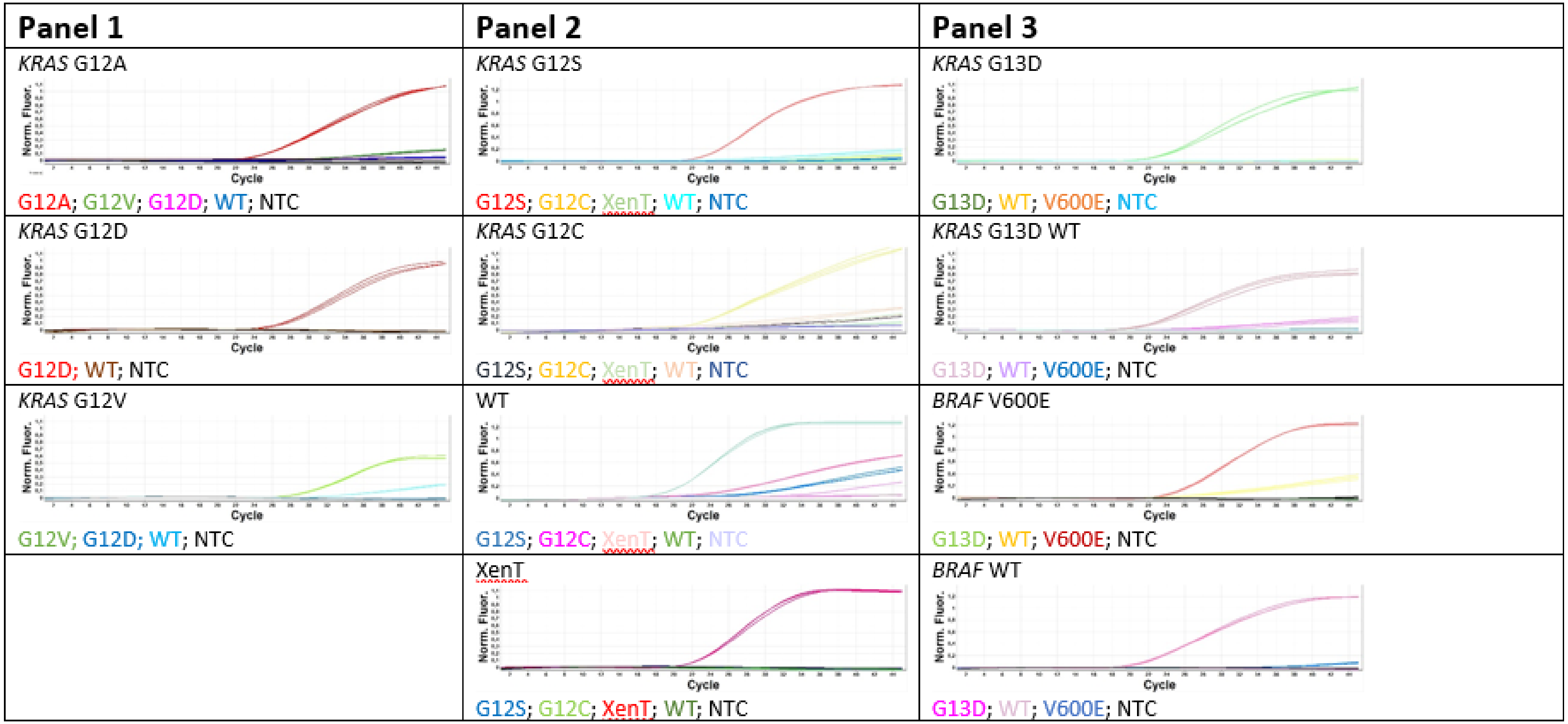

Supplemental figure S 4 *Specifity tests of mediator probes in a qPCR device (RotorgeneQ, Qiagen) in singleplex reactions. Each mediator probe is tested for the targets in the respective 4-plex assay panel.*

5.2. 2-plex MP ddPCR assay LoB and LoD data in Stilla dPCR device

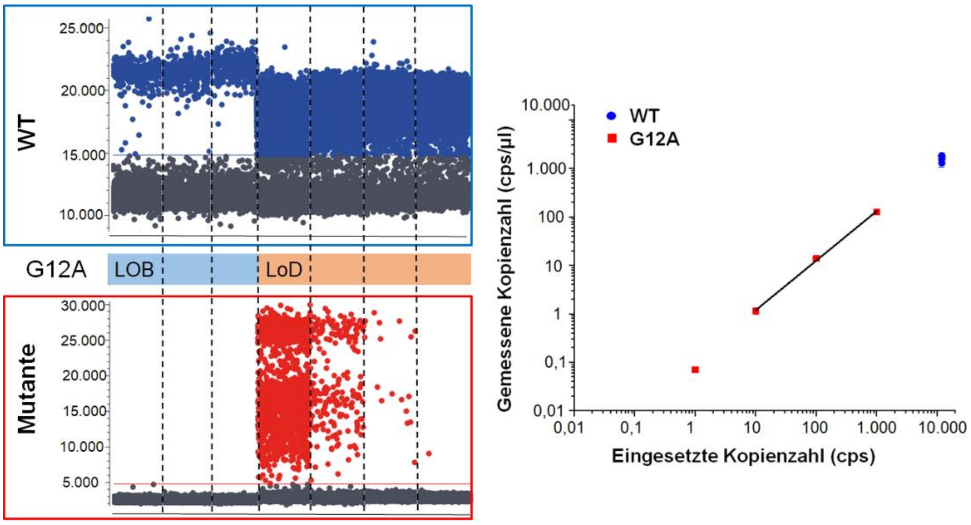

Supplemental figure S 5 *LoB and LoD results with linear fit for 2-plex MP ddPCR assay characterization of KRAS G12A and its corresponding wild type in Stilla system.*

Supplemental table S 7 *LoB and LoD data for 2-plex MP ddPCR assay characterization of KRAS G12A and its corresponding wild type in Stilla system.*

	Mutant (copies/µl)	***# positive droplets mutants***	***WT (copies/µl)***	***# positive droplets WT***	***# droplets***
Plasma eluate	0	0	23.2	317	23,640
Plasma eluate	0	0	27	369	23,468
Plasma eluate	0	0	23.8	314	22,633
NTC	0	0	0.07	1	26,251
1,011 copies/reaction G12A 12,000 copies/reaction WT	125.8	1,819	1,525	15,124	25,598
101 copies/reaction G12A 12,000 copies/reaction WT	13.7	206	1,274	13,555	25,762
10.1 copies/reaction G12A 12,000 copies/reaction WT	1.14	17	1,805	16,569	25,380
1.01 copies/reaction G12A 12,000 copies/reaction WT	0.07	1	1,731	16,540	25,945

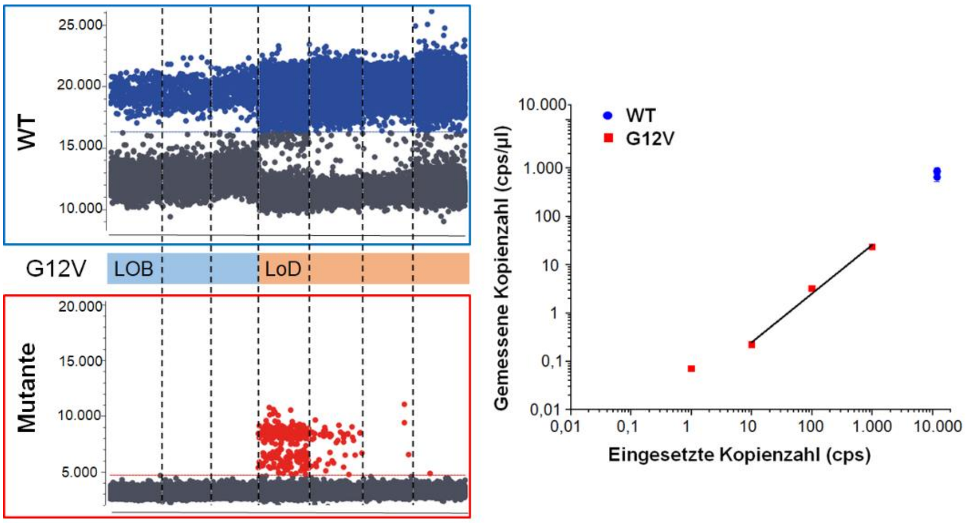

Supplemental figure S 6 *LoB and LoD results with linear fit for 2-plex MP ddPCR assay characterization of KRAS G12V and its corresponding wild type in Stilla system.*

Supplemental table S 8 *LoB and LoD data for 2-plex MP ddPCR assay characterization of KRAS G12V and its corresponding wild type in Stilla system.*

	Mutant (copies/µl)	***# positive droplets mutants***	***WT (copies/µl)***	***# positive droplets WT***	***# droplets***
Plasma eluate	0	0	23.2	331	24,550
Plasma eluate	0	0	24.5	334	23,453
Plasma eluate	0	0	24.9	323	22,262
NTC	0.7	1	0	0	23,193
1,011 copies/reaction G12V 12,000 copies/reaction WT	22.8	321	817.1	9,215	24,221
101 copies/reaction G12V 12,000 copies/reaction WT	3.18	47	874.7	10,134	25,271
10.1 copies/reaction G12V 12,000 copies/reaction WT	0.22	3	638.9	7,406	23,716
1.01 copies/reaction G12V 12,000 copies/reaction WT	0.07	1	637.9	7,743	24,828

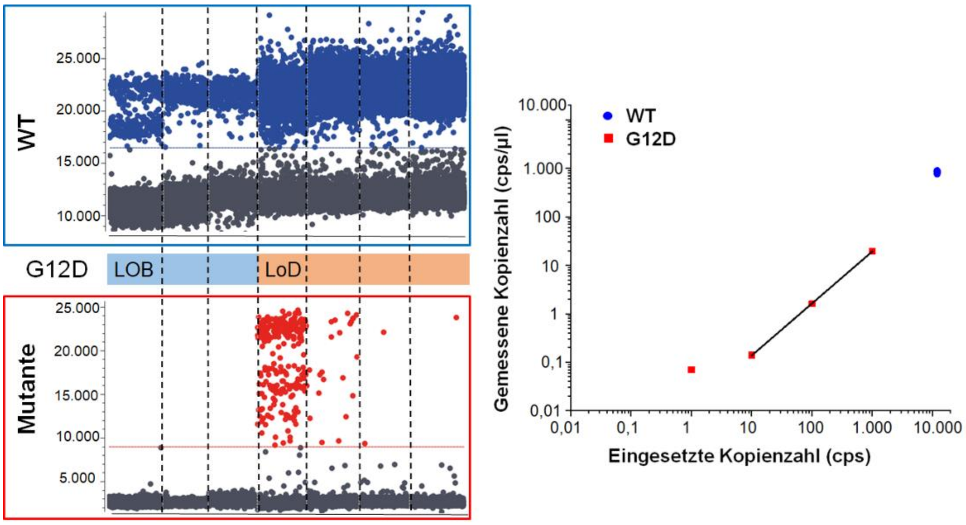

Supplemental figure S 7 *LoB and LoD results with linear fit for 2-plex MP ddPCR assay characterization of KRAS G12D and its corresponding wild type in Stilla system.*

Supplemental table S 9 *LoB and LoD data for 2-plex MP ddPCR assay characterization of KRAS G12D and its corresponding wild type in Stilla system.*

	Mutant (copies/µl)	***# positive droplets mutants***	***WT (copies/µl)***	***# positive droplets WT***	***# droplets***
Plasma eluate	0	0	23.7	347	25,133
Plasma eluate	0	0	27.7	343	21,287
Plasma eluate	0	0	29.4	395	23,156
NTC	0	0	0	0	25,579
1,011 copies/reaction G12D 12,000 copies/reaction WT	19.6	262	833.3	8,871	22,964
101 copies/reaction G12D 12,000 copies/reaction WT	1.6	23	773.2	8,945	24,554
10.1 copies/reaction G12D 12,000 copies/reaction WT	0.14	2	885.5	9,639	23,813
1.01 copies/reaction G12D 12,000 copies/reaction WT	0.07	1	837.9	9,852	25,396

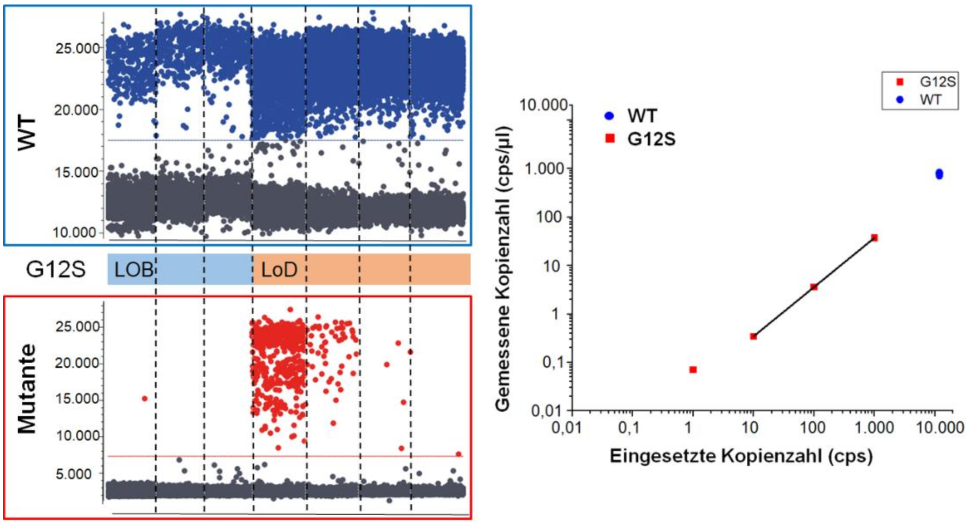

Supplemental figure S 8 *LoB and LoD results with linear fit for 2-plex MP ddPCR assay characterization of KRAS G12S and its corresponding wild type in Stilla system.*

Supplemental table S 10 *LoB and LoD data for 2-plex MP ddPCR assay characterization of KRAS G12S and its corresponding wild type in Stilla system.*

	Mutant (copies/µl)	***# positive droplets mutants***	***WT (copies/µl)***	***# positive droplets WT***	***# droplets***
Plasma eluate	0,07	1	23.9	317	22,828
Plasma eluate	0	0	21.3	291	23,410
Plasma eluate	0	0	25.3	337	22,924
NTC	0	0	0	0	23,288
1,011 copies/reaction G12S 12,000 copies/reaction WT	36.6	540	759.2	9,134	25,437
101 copies/reaction G12S 12,000 copies/reaction WT	3.6	53	781.9	9,243	25,148
10.1 copies/reaction G12S 12,000 copies/reaction WT	0.34	5	823.1	9,650	25,220
1.01 copies/reaction G12S 12,000 copies/reaction WT	0.07	1	699.5	8,512	25,315

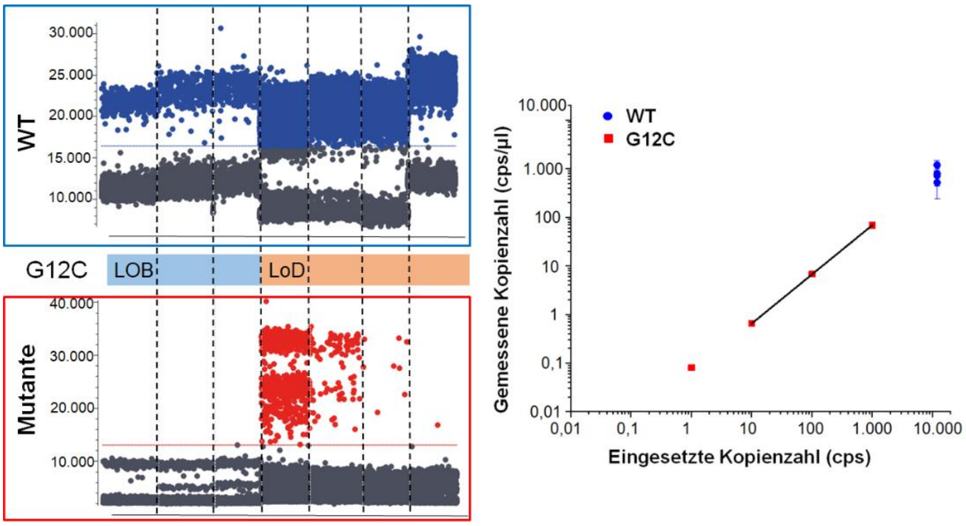

Supplemental figure S 9 *LoB and LoD results with linear fit for 2-plex MP ddPCR assay characterization of KRAS G12C and its corresponding wild type in Stilla system.*

Supplemental table S 11 *LoB and LoD data for 2-plex MP ddPCR assay characterization of G12C and its corresponding wild type in Stilla system.*

	Mutant (copies/µl)	***# positive droplets mutants***	***WT (copies/µl)***	***# positive droplets WT***	***# droplets***
Plasma eluate	0	0	20.6	299	24,916
Plasma eluate	0	0	22.1	318	24,716
Plasma eluate	0	0	25.8	309	20,610
NTC	0	0	0	0	24,656
1,011 copies/reaction G12C 12,000 copies/reaction WT	68.7	851	1,160	10,639	21,569
101 copies/reaction G12C 12,000 copies/reaction WT	6.78	92	790.6	8,605	23,210
10.1 copies/reaction G12C 12,000 copies/reaction WT	0.65	8	719.9	7,187	20,883
1.01 copies/reaction G12C 12,000 copies/reaction WT	0.08	1	359.2	3,986	21,004

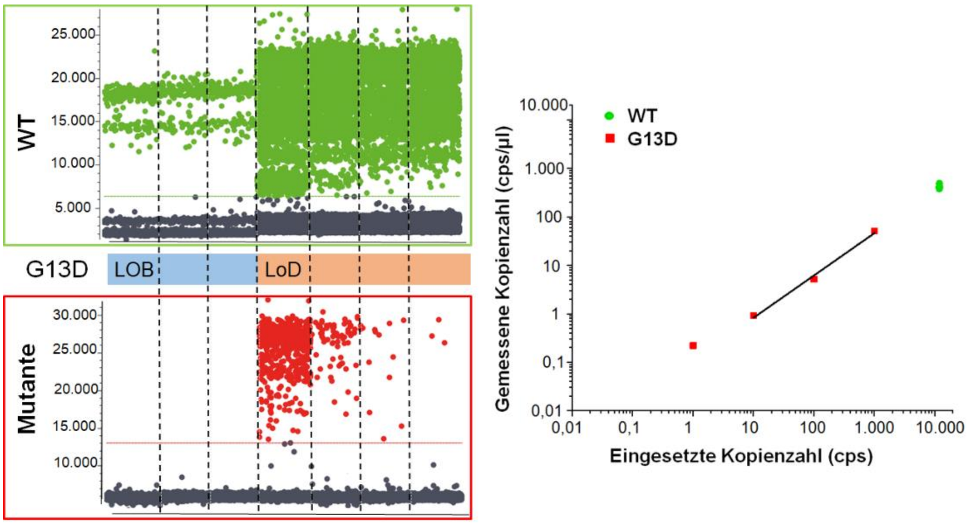

Supplemental figure S 10 *LoB and LoD results with linear fit for 2-plex MP ddPCR assay characterization of KRAS G13D and its corresponding wild type in Stilla system.*

Supplemental table S 12 *LoB and LoD data for 2-plex MP ddPCR assay characterization of KRAS G13D and its corresponding wild type in Stilla system.*

	Mutant (copies/µl)	***# positive droplets mutants***	***WT (copies/µl)***	***# positive droplets WT***	***# droplets***
Plasma eluate	0	0	16.2	235	24,943
Plasma eluate	0	0	13.3	174	22,374
Plasma eluate	0	0	14.6	191	22,443
NTC	0	0	0	0	24,514
1,011 copies/reaction G13D 12,000 copies/reaction WT	50.8	688	483.5	5,790	23,470
101 copies/reaction G13D 12,000 copies/reaction WT	5.16	70	380.9	4,642	23,206
10.1 copies/reaction G13D 12,000 copies/reaction WT	0.91	12	422.9	4,952	22,565
1.01 copies/reaction G13D 12,000 copies/reaction WT	0.22	3	407.2	4,918	23,169

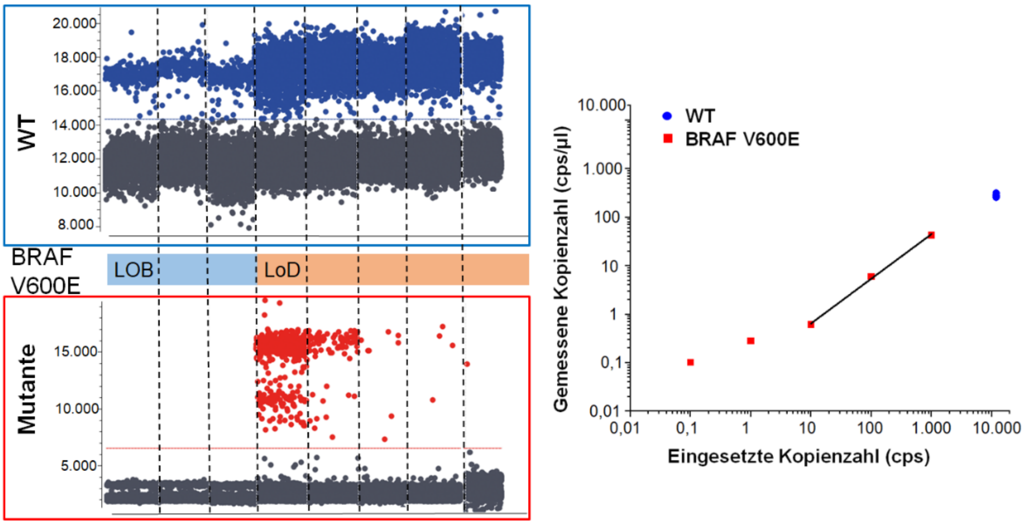

Supplemental figure S 11 *LoB and LoD results with linear fit for 2-plex MP ddPCR assay characterization of BRAF V600E and its corresponding wild type in Stilla system.*

Supplemental table S 13 *LoB and LoD data for 2-plex MP ddPCR assay characterization of BRAF V600E and its corresponding wild type in Stilla system.*

	Mutant (copies/µl)	***# positive droplets mutants***	***WT (copies/µl)***	***# positive droplets WT***	***# droplets***
Plasma eluate	0	0	16.2	225	23,757
Plasma eluate	0	0	13.1	176	22,985
Plasma eluate	0	0	17.2	216	21,495
NTC	0	0	0.07	1	22,953
1,011 copies/reaction V600E 12,000 copies/reaction WT	42.8	575	311.4	3,869	23,198
101 copies/reaction V600E 12,000 copies/reaction WT	5.91	81	276.6	3,503	23,417
10.1 copies/reaction V600E 12,000 copies/reaction WT	0.61	8	281	3,396	22,371
1.01 copies/reaction V600E 12,000 copies/reaction WT	0.28	4	258.6	3,388	24,098
Plasma eluate	0.1	1	255.2	2,478	17,845

5.3. 4-plex MP ddPCR assay LoB and LoD data

Supplemental table S 14 *Allele frequencies of LoB and LoD data for 4-plex MP ddPCR assay characterization with LionheartLX.*

	***Panel 1* - KRAS**			***Panel 2* - KRAS**			***Panel 3***
	**G12V**	**G12A**	**G12D**	**G12C**	**G12S**	***KRAS* G13D**	***BRAF* V600E**
Allele frequency in % ***4-plex MP ddPCR assay***	12.448	3.943	5.942	46.338	1.659	4.280	2.782
	1.100	0.124	0.311	6.385	0.216	0.572	0.574
	0.047	0.009	0.028	0.819	0.062	0.034	0.050
	0.000	0.012	0.024	0.383	0.040	0.048	0.068
R^2 MP ddPCR assays	0.990	0.980	0.980	0.990	0.980	0.990	0.990
LoB in copies/ml plasma	0	0	0	0	0	9.82	16.29

Supplemental table S 15 *LoD copies/reaction data for 4-plex MP ddPCR assay characterization with LionheartLX.*

	***Panel 1* - KRAS**				***Panel 2* - KRAS**				***Panel 3***			
Mutant copies/reaction (WT copies/reaction)	**G12V**	**G12A**	**G12D**	**WT**	**G12C**	**XenT**	**G12S**	***WT***	***KRAS* G13D**	***WT KRAS***	***BRAF* V600E**	***WT BRAF***
1011 (12,000)	35.46	11.23	16.93	284.91	265.18	18.79	9.50	572.27	20.97	489.93	17.13	615.70
101,1 (12,000)	3.82	0.43	1.08	347.44	36.40	1.57	1.23	570.1	2.82	493.55	3.34	581.28
10,11 (12,000)	0.32	0.06	0.19	689.05	4.90	0.43	0.37	598.1	0.18	530.76	0.27	541.60
1,01 (12,000)	0.00	0.07	0.14	591.53	2.07	0.21	0.21	541.17	0.26	536.53	0.43	625.20

Supplemental table S 16 *LoB copies/reaction data for 4-plex MP ddPCR assay characterization with LionheartLX.*

	Panel 1 **- KRAS**				***Panel 2*** **- KRAS**				***Panel 3***			
Eluates	**G12V**	**G12A**	**G12D**	**WT**	**G12C**	**XenT**	**G12S**	***WT***	***KRAS*** **G13D**	***WT KRAS***	***BRAF*** **V600E**	***WT BRAF***
Eluate Pool 1 (copies/reaction)	0.00	0.00	0.00	21.20	0.00	0.00	0.00	19.51	0.00	14.55	0.08	12.65
Eluate Pool 2 (copies/reaction)	0.00	0.00	0.00	22.80	0.00	0.00	0.00	19.42	0.07	12.85	0.07	13.34
Eluate Pool 3 (copies/reaction)	0.00	0.00	0.00	22.80	0.00	0.00	0.00	19.03	0.00	14.80	0.08	11.87
Eluate Pool 4 (copies/reaction)	0.00	0.00	0.00	0.09	0.00	0.00	0.00	0.02	0.00	0.000	0.00	4.70

5.4. MP ddPCR assay performance data with patient samples

Supplemental table S 17 *Data for measurements of patient samples with 2-plex LNA, 2-plex MP ddPCR and 4-plex MP ddPCR assay with Biorad (2-plex LNA), Stilla Naica (2-plex MP ddPCR) and LionheartLX (4-plex MP ddPCR).*

Patient ID	Time point	Gene	Mutation	Panel	2-plex LNA copies/µl eluate	2-plex MP copies/µl eluate	4-plex MP copies/µl eluate
13941	1	*KRAS*	G12D	1	55	46.40	-
13941	2	*KRAS*	G12D	1	5.4	4.85	-
13941	3	*KRAS*	G12D	1	1.62	3.20	-
13941	4	*KRAS*	G12D	1	2.72	4.05	-
13941	2	*BRAF*	V600E	3	0.396	0.39	0.33
13941	3	*BRAF*	V600E	3	0.264	0.33	0.37
13941	7	*BRAF*	V600E	3	0.176	0.39	0.39
13941	2	*BRAF*	WT	3	732.6	668.80	540.65
13941	3	*BRAF*	WT	3	170.28	200.75	108.90
13941	7	*BRAF*	WT	3	97.24	87.45	76.78
13941	1	*KRAS*	WT	1	257.4	295.10	-
13941	2	*KRAS*	WT	1	51.6	58.60	-
13941	3	*KRAS*	WT	1	1246	1831.45	-
13941	4	*KRAS*	WT	1	45.6	57.15	-
23270	1	*KRAS*	G13D	3	820.60	806.30	160.82
23270	1	*KRAS*	WT	3	162.25	159.50	720.50
29722	1	*KRAS*	G13D	3	311.41	156.75	0.00
29722	1	*KRAS*	WT	3	0.00	0.00	151.25
32066	2	*KRAS*	G12D	1	1.58	1.10	1.10
32066	2	*KRAS*	WT	1	420.20	853.60	737.00
49649	1	*KRAS*	G13D	3	555.50	613.25	0.00
49649	2	*KRAS*	G13D	3	73.59	67.65	0.00
49649	1	*KRAS*	WT	3	0.00	0.00	500.50
49649	2	*KRAS*	WT	3	0.00	0.00	67.10
51036	4	*KRAS*	G12D	1	5.06	4.015	4.07
51036	5	*KRAS*	G12D	1	6.16	7.92	11.28
51036	6	*KRAS*	G12D	1	13.86	20.08	18.46
51036	4	*KRAS*	WT	1	7245.30	6572.00	5973.00
51036	5	*KRAS*	WT	1	5082.00	6011.50	5560.50
51036	6	*KRAS*	WT	1	1360.70	1794.10	1485.00
67721	1H	*KRAS*	G12C	2	5.81	5.11	8.84
67721	2G	*KRAS*	G12C	2	4.27	6.57	5.86
67721	4H	*KRAS*	G12C	2	1.41	16.07	21.75
67721	1H	*KRAS*	WT	2	62.04	118.21	114.68
67721	2G	*KRAS*	WT	2	30.36	71.79	69.83
67721	4H	*KRAS*	WT	2	26.40	207.86	166.67

References

1. Schlenker F, Kipf E, Borst N, Hutzenlaub T, Zengerle R, Stetten F von, Juelg P. Virtual Fluorescence Color Channels by Selective Photobleaching in Digital PCR Applied to the Quantification of KRAS Point Mutations. Anal Chem 2021;93:10538–45.
